# Intensive luteal phase support in hormone replacement and modified natural cycle frozen embryo transfers in ovulatory patients: A propensity score-matched study

**DOI:** 10.1371/journal.pone.0327470

**Published:** 2025-07-17

**Authors:** Huong Thi Lien Nguyen, Thang Duc Le, Long Bao Hoang, Anh Thi Tu Phi, Hieu Phuc Nguyen, Quy Quang Luu, Thuy Thu Tran, Tuyen Thi Thanh Nguyen, Huy Tien Nguyen, Phuong Thi Mai Giap, Thuy Le Nguyen, Anh Tuan Cao, Huy Quoc Hoang, Hong Thi Nguyen, Tien Van Do, Jean Noel Hugues, Hoang Le

**Affiliations:** 1 Assisted Reproduction Center, Tam Anh General Hospital, Hanoi, Vietnam; 2 College of Health Sciences, VinUniversity, Hanoi, Vietnam; 3 Department of Obstetrics and Gynecology, Thai Nguyen University of Medicine and Pharmacy, Thai Nguyen, Vietnam; 4 Budapest University of Technology and Economics, Budapest, Hungary; 5 Department of Obstetrics, Gynecology and Reproductive Medicine, Hôpitaux Universitaires Paris Seine Saint-Denis, Assistance Publique-Hôpitaux de Paris, Bondy, France; Université Paris 13, UFR SMBH, Bobigny, France; King Saud University / Zagazig University, EGYPT

## Abstract

**Background:**

The optimal endometrial preparation protocol for frozen embryo transfer (FET) remains controversial, with different cycle regimens and luteal phase support strategies across studies yielding conflicting results. This study aimed to compare the pregnancy outcomes of modified natural cycles (mNC) versus hormone replacement therapy (HRT) cycles, both with intensive luteal support using vaginal micronized progesterone and oral dydrogesterone.

**Methods:**

This retrospective cohort study included 2365 FET cycles (1892 HRT and 473 mNC) in ovulatory women. Both groups received vaginal progesterone (800 mg/day) and oral dydrogesterone (30 mg/day) from the day after ovulation trigger or upon progesterone initiation. Propensity score matching was used to balance baseline characteristics, resulting in 1419 HRT and 473 mNC cycles for analysis. Treatment effect estimates with 95% confidence intervals were estimated using appropriate regression models.

**Results:**

The propensity score-matched population had similar live birth rate (34.7% in the mNC group and 34.8% in the HRT group; aRR 1.02, 95% CI 0.80–1.29), pregnancy rate (54.3% vs 51.3%), clinical pregnancy rate (42.9% vs 42.0%), ongoing pregnancy rate (35.5% vs 35.7%), and miscarriage rate (7.8% vs 7.1%). There were no significant differences in multiple pregnancy rates, gestational age at delivery, birthweight, preterm birth rates between the two protocols.

**Conclusions:**

In ovulatory women undergoing FET with intensive luteal phase support, the use of HRT or mNC for endometrial preparation yields comparable pregnancy and live birth rates.

## Introduction

Frozen embryo transfer (FET), which is fueled by significant advancements in vitrification techniques, coupled with its expanding indications, has emerged as an integral component of assisted reproductive technology (ART) [1,2]. The “freeze-all” strategy can reduce the risk of ovarian hyperstimulation syndrome, enhance endometrial receptivity, and allow for preimplantation genetic testing, all of which have contributed to the rise of FET [3–6]. However, the successful of FET depends on the precise synchronization between embryo developmental stage and endometrial receptivity, which depends on choosing the optimal endometrial preparation protocol [7].

The predominant methods for endometrial preparation in current practice include the natural cycle (NC), modified natural cycle (mNC), and artificial cycle with hormone replacement therapy (HRT). Both the NC and the mNC protocols rely on endogenous hormone production and corpus luteum formation. The mNC approach is considered more flexible due to the active intervention that to promotes the maturation of the dominant follicle. However, the NC and the mNC protocols may lead to a relatively high cycle cancellation rate, up to 20%, reported in some studies, due to the absence of the dominant follicle [8]. It is worth emphasizing that the HRT protocol provides better control over endometrial development by administering estradiol and progesterone externally, it may increase the risks of obstetric and perinatal complications due to the lack of a corpus luteum [9–11].

Despite numerous randomized controlled trials (RCT) comparing the pregnancy outcomes and maternal-fetal safety of these endometrial preparation methods, the superiority of one approach over another remains elusive, mainly due to the heterogeneity in study designs, specifically in monitoring protocols, use of ovulation triggers, and luteal phase support (LPS). A recent systematic review and meta-analysis assessing obstetric and neonatal outcomes following NC and HRT endometrial preparation, encompassing 30 studies with over 110,000 patients, revealed that the NC group receiving LPS had a lower risk of preterm birth compared to the HRT group while NC without LPS did not. However, the interpretation of these findings could not lead to a definite conclusion due to the substantial heterogeneity across studies and the low to moderate quality of evidence [12].

In HRT cycles, the sole reliance on vaginal micronized progesterone may result in suboptimal absorption and lower serum progesterone concentrations in a subset of patients, with detrimental effects on pregnancy outcomes following FET [13,14]. To circumvent this drawback, it has been advocated to monitor serum progesterone prior to embryo transfer. Rescue protocols involving additional progesterone administration in patients with low levels has been shown to restore pregnancy rates similar to those of patients with adequate progesterone levels [15,16]. Concurrently, some evidence came from mNC cycles that clinical pregnancy rates could be improved by a supplementation with exogenous progesterone [17,18].

Given the potential role of intensive LPS in optimizing the endometrial milieu and pregnancy outcomes, the question arises whether the clinical effectiveness of HRT and mNC protocols would still differ significantly if both incorporated vaginal micronized progesterone and oral dydrogesterone for LPS. To address this issue, we conducted a retrospective cohort study to compare the pregnancy outcomes of HRT and mNC FET cycles in ovulatory women who received intensive LPS.

## Materials and methods

### Study design and participants

A retrospective analysis of all autologous FET cycles performed between January 2022 and September 2023 was carried out at our academic fertility center. Women aged 20–45 years with regular ovulatory cycles, defined as a menstrual cycle length of 24–38 days, who underwent endometrial preparation with either an HRT or mNC protocol were eligible for inclusion. The exclusion criteria were: oocyte vitrification, adenomyosis, submucosal fibroids, surgically retrieved sperm, hydrosalpinx documented on hysterosalpingography, and endometrial thickness <7mm at the final ultrasound assessment prior to progesterone initiation. Patients’ baseline characteristics, treatment parameters, and pregnancy outcomes were extracted from the electronic medical records. For patients receiving prenatal care and delivery at outside facilities, obstetric and neonatal outcomes were obtained through standardized telephone interviews. The study was approved by the Institutional Review Board on August 29, 2024 (IRB reference number: IRB.TAHN.071). Data were accessed for research purposes on August 30, 2024. All patient data were anonymized prior to analysis, and the authors did not have access to any identifying information during or after data collection.

### Endometrial preparation and luteal support

Following baseline transvaginal ultrasound on cycle days 2–4 and counseling regarding endometrial preparation options, the selection of the HRT or mNC protocol was determined through a shared decision-making process involving both the patient and the treating physician, rather than being based on rigid, pre-defined clinical criteria. This decision was primarily guided by non-clinical factors and specific patient preferences. Key considerations encompassed logistical aspects, such as patient convenience related to the frequency and feasibility of monitoring visits (particularly relevant for mNC cycles), patient desire concerning the extent of exogenous hormone administration, and individual acceptance of the potential risk for cycle cancellation inherent to the mNC protocol.

In the mNC protocol, ultrasound monitoring was started on cycle day 7, with subsequent visits scheduled according to follicular growth. When the leading follicle reached a mean diameter of ≥16 mm with an endometrial thickness ≥7 mm and serum progesterone level <1.5 ng/ml, final oocyte maturation was triggered with 5000 IU of human chorionic gonadotropin (hCG, IVF-C, LG Chem). Progesterone supplementation was initiated the following day. Cycles were cancelled if no dominant follicle developed by day 21, premature ovulation occurred, or serum progesterone level was ≥ 1.5 ng/ml.

In the HRT protocol, oral estradiol valerate 6 mg daily (Progynova, Bayer) was started on cycle day 2–4. An ultrasound follow-up was performed on day 10 to assess endometrial development, with dose adjustments if needed. Progesterone supplementation was initiated on day 14 if the endometrium reached a minimum thickness of 7 mm. Cycles were cancelled if the endometrial thickness remained below 7 mm despite extending estrogen administration to 21 days. In pregnant patients, estrogen was continued until 7 weeks of gestation.

Two groups received an identical intensive LPS regimen including vaginal micronized progesterone 800 mg daily (Cyclogest, Actavis) and oral dydrogesterone 30 mg daily (Duphaston, Abbott). Serum progesterone and estradiol levels were not routinely monitored during the luteal phase in either protocol. This approach was based on the fixed, high-dose nature of our combined LPS regimen, which aimed to provide sufficient support without the need for individualized hormone level adjustments or rescue protocols, consistent with recent perspectives suggesting that such monitoring may be unnecessary with adequate LPS [19]. Cleavage-stage embryos were transferred on the morning of progesterone day 4, while blastocysts were transferred on the morning of progesterone day 6. Serum beta-hCG levels were checked 10–12 days after transfer, followed by transvaginal ultrasound 2 weeks later to confirm fetal viability. LPS was maintained until 12 weeks of gestation in ongoing pregnancies.

### Embryo evaluation and selection

Following oocyte retrieval, oocyte-cumulus complexes were cultured for 2–4 hours, then denuded and assessed for maturity. Metaphase II oocytes underwent ICSI, with the injected oocytes cultured in a continuous single culture medium (Fujifilm Irvine Scientific) at 37°C, 5% O2, and 6% CO2. Embryo morphology was evaluated on day 3 (67–69 hours post-ICSI), with good-quality cleavage-stage embryos defined as having ≥6 cells, < 25% fragmentation, stage-specific cell sizes, and/or evidence of compaction [20]. Blastocysts were graded on day 5 (114–116 hours post-ICSI) using the Gardner system based on expansion, inner cell mass, and trophectoderm. Blastocysts graded ≥3BB were considered as good quality [21]. Slower growing embryos were cultured until day 6. Embryos were cryopreserved at either cleavage or blastocyst stage using vitrification (Cryotech, Japan). The number of embryos transferred followed the American Society for Reproductive Medicine guidelines [22], considering factors like age, embryo quality, and previous IVF outcomes, with a maximum of 2 embryos transferred per cycle.

### Outcome measures

The primary outcome was the live birth rate, defined by the number of deliveries of at least one live neonate beyond 23 weeks of gestation per embryo transfer cycle. Secondary outcomes included the rates of positive hCG (serum beta-hCG > 5 IU/L at 10–12 days post-transfer), clinical pregnancy (gestational sac on ultrasound), ongoing pregnancy (fetal cardiac activity at 12 weeks), implantation (number of gestational sacs divided by the number of embryos transferred), biochemical pregnancy loss (positive hCG that failed to progress to clinical pregnancy), miscarriage (pregnancy loss after clinical confirmation up to 22 weeks of gestation), and multiple pregnancy (≥2 gestational sacs visualized). The preterm birth rate was defined as the number of deliveries before 37 weeks of gestational age divided by the total number of live births (excluding 5 cases due to missing date of birth information). Other pertinent perinatal outcomes included gestational age at birth, birthweight, and the incidence of major congenital abnormalities.

### Statistical analysis

Baseline characteristics and outcomes were compared between participants who received the HRT and mNC protocol, with categorical variables presented as number (percentage) and quantitative variables presented as means [standard deviation (SD)]. We utilized the propensity score methods to create matched data that achieved balance between the two groups. The propensity score was estimated using a multivariable logistic regression on the baseline characteristics of the participants, including age, body mass index (BMI) group (underweight: < 18, normal BMI: 18– < 23, overweight: 23–25, and obese: > 25 kg/m2), history of cesarean section, number of consecutive failed embryo transfers, duration and type of infertility, number, stage, and quality of embryos transferred. Then we used the MatchIt package for 1:3 matching, considering both optimal and nearest neighbor methods with a caliper of 0.1. The greater reduction in the absolute standardized mean differences (SMD) of the covariates before and after adjustment was achieved with the optimal method (S1-S2 Figures).

The treatment effect of using mNC compared to HRT on clinical outcomes was estimated both unadjusted and adjusted for all covariates that we used in the logistic (propensity scores) model, to clean up possible residual confounding and improve the precision of the estimation. We performed analyses on pregnancy, biochemical pregnancy, clinical pregnancy, ongoing pregnancy, live birth, multiple pregnancy, and implantation rate. For the adjusted analyses, we used the multiple regression model (log-binomial regression to estimate the risk ratio, and the Poisson regression for implantation rate to estimate the mean ratio.

All statistical tests were two-tailed, with P < 0.05 denoting significance. All analyses were performed using the R language version 4.3.1 (R Foundation for Statistical Computing, Vienna, Austria).

## Results

We collected a total of 2365 autologous FET cycles, comprising 1892 HRT and 473 mNC cycles, among which 1419 HRT and 473 mNC cycles were included in the final analysis after the propensity score matching. The patient demographic and cycle characteristics before and after propensity score matching are presented in Table 1. Prior to matching, there were significant differences between the two groups in terms of BMI, and the history of cesarean section and endometrial thickness. After matching, the baseline covariates were well balanced, as evidenced by the lack of remarkable differences between the matched groups and the standardized mean differences falling below 0.1 for all variables (Table 1 and S1-S2 Figures).

**Table 1:** Patient and cycle characteristics before and after propensity score matching.

	Before matching			After matching		
	HRT (n = 1892)	mNC (n = 473)	p	HRT (n = 1419)	mNC (n = 473)	p
Age (years)	33.76 (5.02)	33.91 (4.92)	0.573	33.84 (5.04)	33.91 (4.92)	0.808
Age group (%)			0.444			0.479
* <30*	422 (22.3)	92 (19.5)		315 (22.2)	92 (19.5)	
* 30 to <35*	618 (32.7)	155 (32.8)		450 (31.7)	155 (32.8)	
* 35 to <40*	584 (30.9)	161 (34.0)		444 (31.3)	161 (34.0)	
* 40–45*	268 (14.2)	65 (13.7)		210 (14.8)	65 (13.7)	
BMI (kg/m^2^)	21.50 (2.53)	21.15 (2.35)	0.006	21.25 (2.29)	21.15 (2.35)	0.404
Infertility factor (%)			0.147			0.238
* Male*	1020 (53.9)	230 (48.6)		745 (52.5)	230 (48.6)	
* Female*	95 (5.0)	21 (4.4)		80 (5.6)	21 (4.4)	
* Both male and female*	104 (5.5)	29 (6.1)		83 (5.8)	29 (6.1)	
* Other factors*	673 (35.6)	193 (40.8)		511 (36.0)	193 (40.8)	
Type of infertility (%)						
* Primary*	628 (33.2)	162 (34.2)	0.703	507 (35.7)	162 (34.2)	0.598
* Secondary*	1264 (66.8)	311 (65.8)		912 (64.3)	311 (65.8)	
Duration of infertility (years)	4.62 (3.63)	4.47 (3.71)	0.435	4.49 (3.57)	4.47 (3.71)	0.916
History of cesarean section	487 (25.7)	146 (30.9)	0.028	421 (29.7)	146 (30.9)	0.664
Number of consecutive failed embryo transfers (%)			0.483			0.917
* 0-1*	1261 (66.6)	303 (64.1)		907 (63.9)	303 (64.1)	
* 1-2*	513 (27.1)	135 (28.5)		414 (29.2)	135 (28.5)	
* 3 or more*	118 (6.2)	35 (7.4)		98 (6.9)	35 (7.4)	
Endometrial thickness (mm)	9.47 (1.27)	9.64 (1.38)	0.014	9.44 (1.27)	9.64 (1.38)	0.006
Number of embryos transferred (%)			0.695			1.000
* 1 embryo*	1324 (70.0)	326 (68.9)		979 (69.0)	326 (68.9)	
* 2 embryos*	568 (30.0)	147 (31.1)		440 (31.0)	147 (31.1)	
Good-quality embryos transferred (%)			0.648			0.977
* 0*	463 (24.5)	107 (22.6)		319 (22.5)	107 (22.6)	
* 1*	1283 (67.8)	326 (68.9)		984 (69.3)	326 (68.9)	
* 2*	146 (7.7)	40 (8.5)		116 (8.2)	40 (8.5)	
Type of embryo (%)			0.286			0.964
* Cleavage*	557 (29.4)	154 (32.6)		455 (32.1)	154 (32.6)	
* Blastocyst without PGT-A*	913 (48.3)	210 (44.4)		640 (45.1)	210 (44.4)	
* Euploid blastocyst*	422 (22.3)	109 (23.0)		324 (22.8)	109 (23.0)	

Data are presented as mean (SD) for continuous variables and n (%) for categorical variables. BMI: body mass index; PGT-A: preimplantation genetic testing for aneuploidy.

Table 2 summarizes the pregnancy outcomes of the propensity-matched cohorts. The live birth rate was similar between the mNC and HRT groups (34.7% and 34.8%, respectively; adjusted RR 1.02, 95% CI 0.80–1.29, p = 1.00). Also, there were no significant differences in the secondary outcomes including positive hCG (54.3% vs 51.3%, p = 0.276), clinical pregnancy (42.9% vs 42.0%, p = 0.767), ongoing pregnancy (35.5% vs 35.7%, p = 1.000), implantation (35.2% vs 35.6%, p = 0.859), biochemical pregnancy (11.4% vs 9.3%, p = 0.212), miscarriage (7.8% vs 7.1%, P = 0.683), and multiple pregnancy (4.0% vs 2.7%, p = 0.218) rates.

**Table 2:** Pregnancy outcomes of the propensity score-matched cohorts.

	HRT (n = 1419)	mNC (n = 473)	p
Pregnancy	728 (51.3)	257 (54.3)	0.276
Biochemical pregnancy	132 (9.3)	54 (11.4)	0.212
Clinical pregnancy	596 (42.0)	203 (42.9)	0.768
Ongoing pregnancy	506 (35.7)	168 (35.5)	1.000
Live birth	494 (34.8)	164 (34.7)	1.000
Singleton live birth	463 (93.7)	146 (89.0)	0.07
Twin live birth	31 (6.3)	18 (11.0)	
Implantation rate	865/2460 (35.2)	221/620 (35.6)	0.859
Miscarriage	101 (7.1)	37 (7.8)	0.683
Multiple pregnancy	39 (2.7)	19 (4.0)	0.218
Major birth defect	2 (<1)	1 (<1)	–
Gestational age at birth (weeks)	38.08 (1.81)	37.95 (2.03)	0.453
Preterm birth, singleton	35 (7.6)	8 (5.5)	0.499
Preterm birth, twins	17 (54.8)	7 (38.9)	0.435
Birth weight, singleton (gram)	3170.89 (466.24)	3159.08 (436.53)	0.780
Birth weight, twins (gram)	2432.26 (360.51)	2458.33 (330.44)	0.798

Data are presented as n (%) for categorical variables, mean (SD) for continuous variables, and fractions (%) for rates.

The incidence of major congenital anomalies was low and similar in both arms, with 1 case (<1%) in the mNC group and 2 cases (<1%) in the HRT group. Reported birth defects included a solitary testicle in the mNC group and 2 cardiac malformations (1 pulmonary artery anomaly and 1 case of mitral valve insufficiency) in the HRT group. The frequencies of preterm birth were also low and comparable between the two protocols for both singleton and twin pregnancies (Table 2). Mean birth weights were in the normal range and did not differ significantly for singletons (3159 gram vs 3171 gram) or twins (2458 gram vs 2432 gram) in the mNC and HRT groups, respectively.

**Table 3:** Crude and adjusted relative risks for pregnancy outcomes.

	Unadjusted RR (95% CI)	Adjusted RR (95% CI)
Pregnancy	1.06 (0.96, 1.17)	1.18 (0.93, 1.49)
Biochemical pregnancy	1.23 (0.91, 1.66)	1.27 (0.90, 1.79)
Clinical pregnancy	1.02 (0.91, 1.15)	1.07 (0.85, 1.35)
Ongoing pregnancy	1.00 (0.87, 1.15)	1.02 (0.80, 1.29)
Live birth	1.00 (0.86, 1.15)	1.02 (0.80, 1.29)
Implantation rate	1.02 (0.90, 1.16)	1.04 (0.93, 1.17)
Miscarriage	1.10 (0.76, 1.58)	1.11 (0.75, 1.66)
Multiple pregnancy rate	1.46 (0.85, 2.51)	1.67 (0.90, 3.11)

There was no significant difference in pregnancy outcomes between the mNC and HRT groups, both before and after matching. In the unadjusted analysis, the mNC protocol had a relative risk of 1.00 (95% CI 0.86–1.15) for live birth, 1.02 (95% CI 0.91–1.15) for clinical pregnancy, 1.00 (95% CI 0.87–1.15) for ongoing pregnancy, and 1.02 (95% CI 0.90–1.16) for implantation relative to the HRT protocol. After matching, the corresponding adjusted relative risks remained virtually unchanged at 1.02 (95% CI 0.80–1.29) for live birth, 1.07 (95% CI 0.85–1.35) for clinical pregnancy, 1.02 (95% CI 0.80–1.29) for ongoing pregnancy, and 1.04 (95% CI 0.93–1.17) for implantation (Table 3).

## Discussion

In this large retrospective cohort study using propensity score matching, we found that mNC and HRT protocols with intensive LPS resulted in equivalent pregnancy and live birth outcomes. Among well-matched patient cohorts, the live birth rate was 34.7% in the mNC group and 34.8% in the HRT group. The rates of pregnancy, clinical pregnancy, ongoing pregnancy, implantation, pregnancy loss, multiple pregnancy, gestational age at delivery, birthweight, preterm birth rates were also similar between the two protocols. Our findings suggest that with sufficient luteal phase support, the choice of endometrial preparation for frozen embryo transfer in ovulatory women can be individualized based on patient preference and convenience without compromising outcomes.

These findings are consistent with a recent RCT by Ho et al. (2024) in 1,428 Vietnamese women, which reported similar live birth rates after one FET cycle in the mNC (33%) and HRT (34%) groups, with the notable difference being that the mNC group did not receive progesterone for LPS, and the HRT group used micronized progesterone alone [8]. This contrasts with our study, which employed a combination of vaginal micronized progesterone and oral dydrogesterone in both groups. Our strategy was based on recent evidence regarding optimizing LPS. In HRT cycles, Labarta et al. (2021) demonstrated that low serum progesterone levels on the day of embryo transfer were associated with decreased clinical pregnancy rates when only vaginal progesterone was used [14]. Furthermore, the MIDRONE study also showed that luteal phase support with oral dydrogesterone added to vaginal progesterone led to a higher live birth rate and lower miscarriage rate compared with vaginal progesterone alone [23]. In mNC cycles, the necessity and optimal regimen for LPS remain debated due to the presence of an endogenous corpus luteum. While several small randomized trials and meta-analyses suggest improved clinical pregnancy rates with vaginal progesterone compared to no supplementation [17,18], the rationale for intensive support as used in our study warrants discussion. Existing evidence indicates significant variability in endogenous progesterone levels during the natural luteal phase; for instance, Saupstad et al. (2024) reported a wide range (4.9–91.8 nmol/L) on transfer day in mNC cycles, which did not correlate with clinical pregnancy rates [24]. Currently, there is no established optimal progesterone threshold in mNC, making it difficult to identify patients who might benefit from supplementation. Therefore, providing uniform, intensive LPS to all mNC patients, as done in our study and reflecting common practice in some regions, serves two purposes: 1) it ensures luteal sufficiency even for those with potentially lower endogenous production, and 2) it standardizes the luteal environment between the mNC and HRT groups, thereby isolating the comparison to the endometrial preparation method itself rather than confounding it with differing progesterone levels. Therefore, while Ho et al. demonstrated comparable outcomes between mNC *without* LPS and HRT with vaginal progesterone alone, our study addresses a different but equally relevant clinical scenario by comparing these protocols under conditions where *both* receive standardized, intensive luteal support, potentially mitigating concerns about insufficient endogenous or exogenous progesterone in either arm under less intensive regimens.

Our results stand in contrast to two recent retrospective cohort studies that also employed propensity score matching to compare mNC and HRT protocols. Sun et al. (2024) reported significantly higher live birth (48.1% vs 41.2%) and clinical pregnancy (58.3% vs 51.1%) rates in the mNC group compared to the HRT group [25]. Similarly, Wang et al. (2023) found a higher probability of healthy live birth (35.8% vs 30.6%), and lower risks of pregnancy loss and hypertensive disorders of pregnancy with the mNC protocol [26]. This discrepancy may be explained by the lower progesterone doses used for LPS in the HRT groups of those studies. Whereas 800 mg/day vaginal progesterone plus 30 mg/day dydrogesterone were prescribed for both groups in our protocol, the studies by Wang et al. and Sun et al. used lower doses of vaginal progesterone (200 mg/day) with either oral dydrogesterone (20 mg twice daily) [26] or intramuscular progesterone (60 mg) to transform the endometrium, followed by 10 mg/day of oral dydrogesterone the next day and further increase to 30 mg/day after three days [25].

Importantly, our finding that high-dose progesterone supplementation in the mNC group yielded outcomes comparable to the HRT group challenges concerns about potential deleterious effects of supraphysiologic progesterone levels. Our results align with those of Saupstad et al. (2024) and others, suggesting that within a wide range, elevated progesterone levels in mNC cycles (whether endogenous or supplemented) do not negatively impact clinical pregnancy rates [24]. The similar rates of pregnancy, biochemical pregnancy loss, and miscarriage observed between the mNC and HRT groups in our study further support the notion that early exposure to intensive progesterone support, as provided, does not adversely impact embryo implantation or early development. This provides reassurance regarding the safety of adopting intensive LPS strategies in mNC protocols if deemed clinically appropriate. While our study was not designed to determine the absolute necessity of LPS in mNC or identify subgroups who might better benefit, it demonstrates that an intensive regimen is not detrimental compared to the same regimen in HRT cycles.

Moreover, the low and comparable incidence of congenital malformations in both groups provides reassuring evidence for the safety of oral dydrogesterone use in early pregnancy, corroborating the findings of a recent meta-analysis that reported no increased risk of birth defects with this progestin compared to population-based estimates [27]. These findings lend support to the concept of potentially using intensive LPS (like the combination of vaginal micronized progesterone and oral dydrogesterone) in both HRT and potentially mNC cycles without routine blood monitoring. This approach, consistent with suggestions by Lawrenz et al. (2024) for HRT cycles, could simplify clinical workflow and reduce patient burden [19].

The major strengths of our study include the large sample size, the use of the propensity score matching to minimize selection bias and confounding, and the homogeneity of the luteal support protocol. With over 2300 FET cycles included in the analysis, our study is nowadays the largest set up to compare mNC and HRT protocols in the setting of intensive LPS. The propensity score matching created well-balanced cohorts with standardized mean differences <0.1 for all baseline covariates, allowing valid comparisons.

However, the study has some limitations. First, due to the retrospective design, we could not fully control for all potential unmeasured confounders, although we applied strict exclusion criteria and performed multivariable analyses to mitigate their impact. Second, as previously mentioned, the choice of the endometrial preparation protocol was not randomized but resulted from the discretion of the treating physician and patient preferences, so confounding bias, despite PSM, could not be entirely controlled. Third, this study was conducted at a single academic fertility center, which may limit the generalizability of the findings to other populations or settings with different patient demographics or clinical practices. While the internal validity is strengthened by the large sample size and rigorous methodology, the external validity of our study can be limited compared to multi-center studies involving more diverse populations. Finally, a significant limitation relates to the collection of data on obstetric outcomes, particularly pregnancy complications like preeclampsia and gestational diabetes. For patients who delivered at outside facilities, these data were obtained via self-report during telephone interviews. We recognize that this method is prone to recall bias and potential inaccuracies compared to data extracted directly from medical records, especially for complex diagnoses requiring specific clinical criteria. Given these concerns about data reliability for such complications, we decided not to include these variables in the main comparative analysis to maintain the integrity of our primary findings. While data on core outcomes like live birth, miscarriage, preterm birth, and birth weight were considered more reliably reported, the absence of robust data on critical complications like preeclampsia is a notable omission, particularly given the ongoing debate about differential risks between HRT (lacking a corpus luteum) and mNC cycles. Although the expected incidence of these complications might be low in our relatively young, healthy population, potentially limiting statistical power even with complete data, future research ideally utilizing direct medical record abstraction is necessary to comprehensively evaluate the impact of these protocols on the full spectrum of obstetric outcomes.

## Conclusion

Our results suggest that in patients receiving intensive LPS with a combination of vaginal micronized progesterone and oral dydrogesterone, the clinical effectiveness of FET with HRT and mNC protocols is equivalent. The choice of endometrial preparation can be based on individual patient characteristics and preferences.

## Supporting information

S1 Fig Kernel density estimate plot of propensity scores. The propensity scores, which represent the probability of being treated with the modified natural cycle (mNC) protocol for each embryo transfer cycle, were estimated using a multivariable logistic regression model. This model predicted the likelihood of selecting the mNC protocol based on patient and cycle characteristics, including age, body mass index, history of cesarean section, number of consecutive failed embryo transfers, duration and type of infertility, number, stage, and quality of embryos transferred. The plot shows the distribution of propensity scores in the hormone replacement therapy (HRT) group and the mNC group before and after propensity score matching.
(TIF)

S2 Fig A Love plot evaluating the balance after two propensity score matching methods. The plot compares the absolute standardized mean differences of covariates between the modified natural cycle (mNC) and hormone replacement therapy (HRT) groups after propensity score matching using two different methods: optimal and nearest neighbor (with a caliper of 0.1). Each dot represents a covariate. The optimal matching method (red dots) achieved better balance compared to the nearest neighbor method (green dots), as evidenced by the smaller absolute standardized mean differences across all covariates. The dashed vertical lines indicate the recommended thresholds for acceptable balance (0.1 and 0.2).
(TIF)

## Abbreviations

FET: Frozen embryo transfer
ART: Assisted reproductive technology
NC: Natural cycle
mNC: Modified natural cycle
HRT: Hormone replacement therapy
LPS: Luteal phase support
RCT: Randomized controlled trial
BMI: Body mass index
PGT-A: Preimplantation genetic testing for aneuploidy
ICSI: Intracytoplasmic sperm injection
hCG: Human chorionic gonadotropin
SD: Standard deviation
SMD: Standardized mean difference
RR: Relative risk
CI: Confidence interval.
