# Supplementary figures and images for: Intensive luteal phase support in hormone replacement and modified natural cycle frozen embryo transfers in ovulatory patients: A propensity score-matched study
Source: PLoS One. 2025 Jul 17;20(7):e0327470. doi: 10.1371/journal.pone.0327470 (PMC12270165; doi:10.1371/journal.pone.0327470)

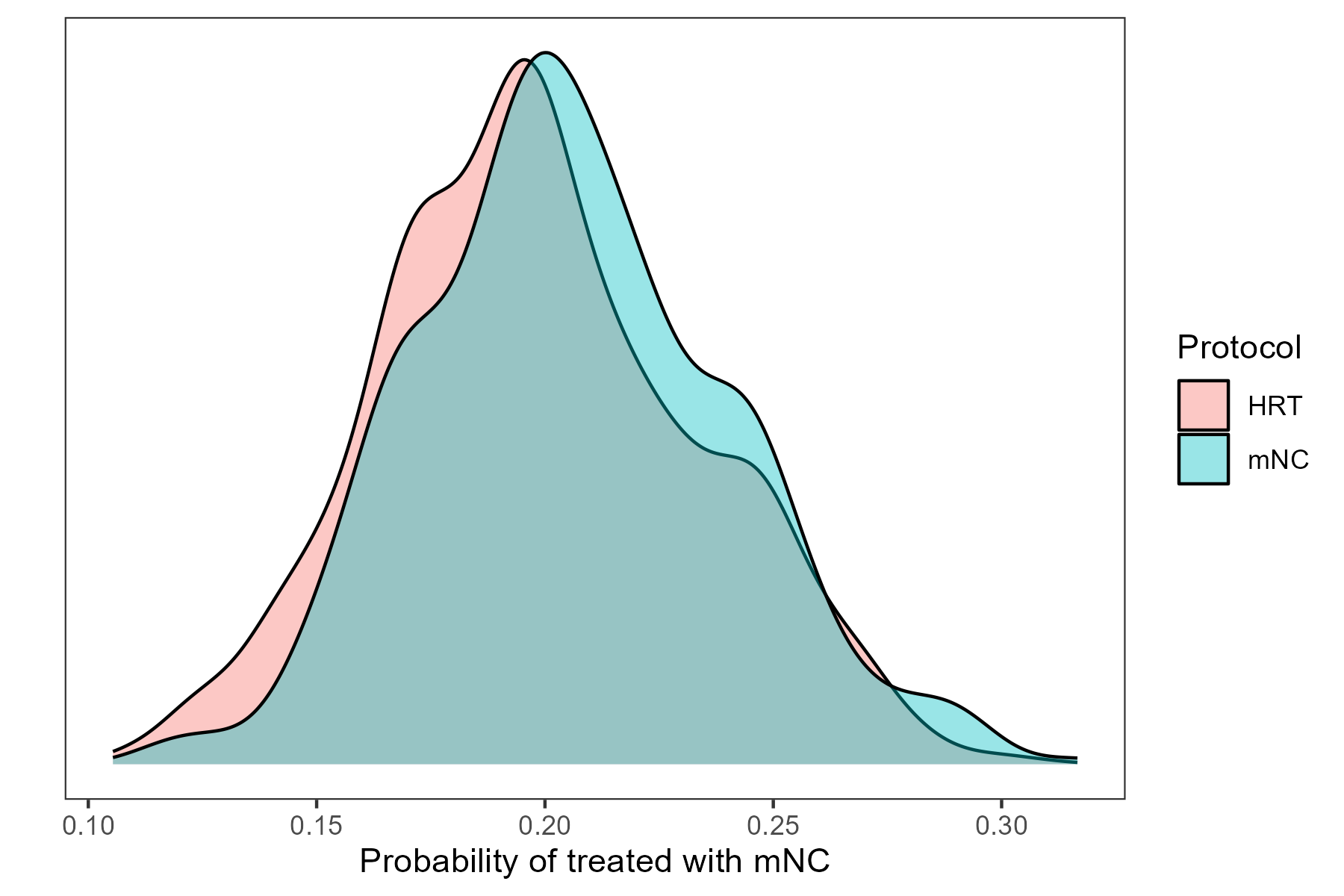

Supplement: S1 Fig — The propensity scores, which represent the probability of being treated with the modified natural cycle (mNC) protocol for each embryo transfer cycle, were estimated using a multivariable logistic regression model. This model predicted the likelihood of selecting the mNC protocol based on patient and cycle characteristics, including age, body mass index, history of cesarean section, number of consecutive failed embryo transfers, duration and type of infertility, number, stage, and quality of embryos transferred. The plot shows the distribution of propensity scores in the hormone replacement therapy (HRT) group and the mNC group before and after propensity score matching. (TIF) [file pone.0327470.s001.tif]

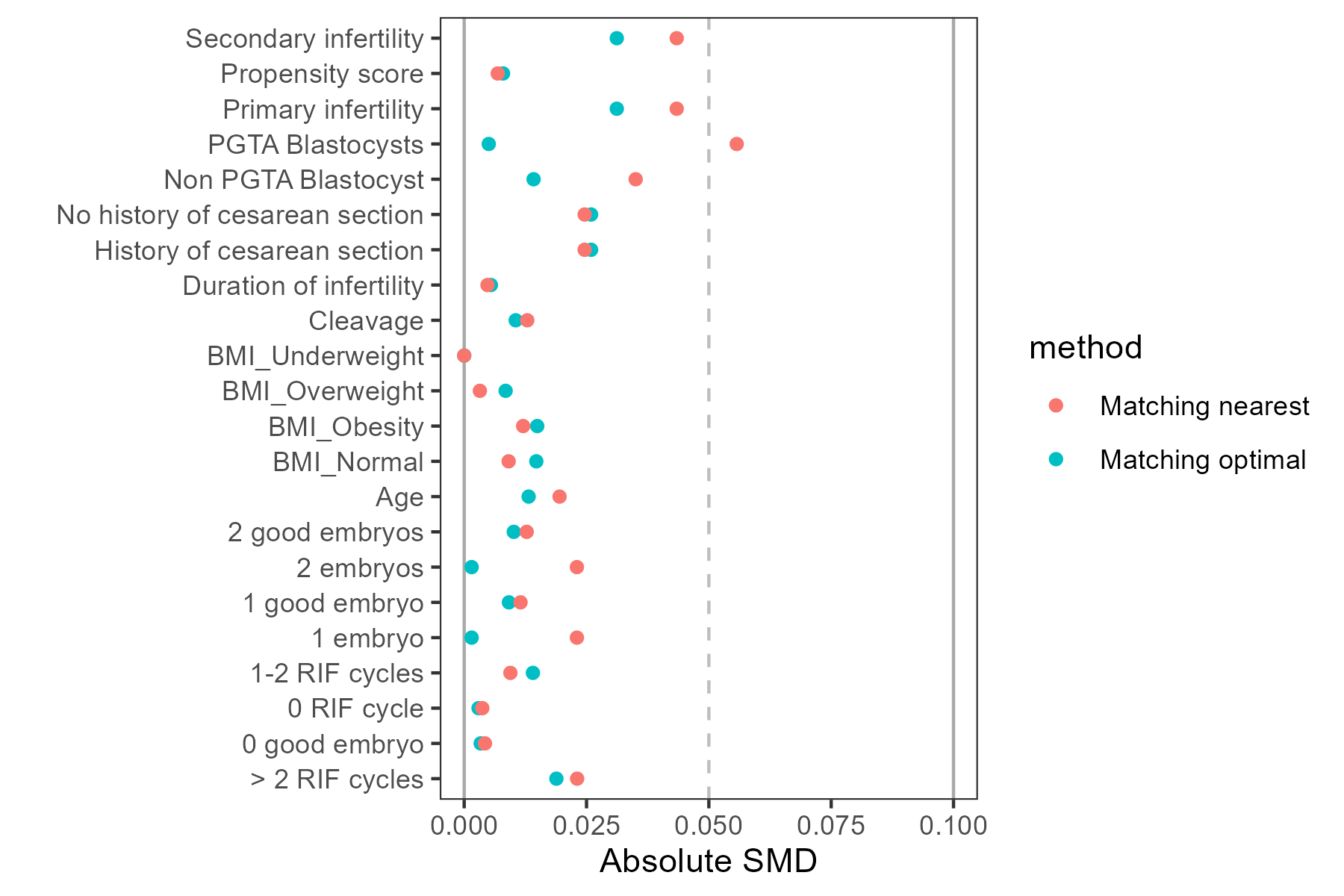

Supplement: S2 Fig — The plot compares the absolute standardized mean differences of covariates between the modified natural cycle (mNC) and hormone replacement therapy (HRT) groups after propensity score matching using two different methods: optimal and nearest neighbor (with a caliper of 0.1). Each dot represents a covariate. The optimal matching method (red dots) achieved better balance compared to the nearest neighbor method (green dots), as evidenced by the smaller absolute standardized mean differences across all covariates. The dashed vertical lines indicate the recommended thresholds for acceptable balance (0.1 and 0.2). (TIF) [file pone.0327470.s002.tif]
